# A MIXED-METHODS EVALUATION OF LAY COACH-SUPPORTED EMPOWER@HOME FOR GERIATRIC DEPRESSION

**DOI:** 10.1093/geroni/igae098.0960

**Published:** 2024-12-31

**Authors:** Xiaoling Xiang

**Affiliations:** University of Michigan, Ann Arbor, Michigan, United States

## Abstract

Empower@home is a novel internet-based cognitive behavioral therapy (iCBT) program specifically designed to reduce depression in older adults. It features 9 online lessons and a physical workbook, complemented by optional calls from lay coaches. A previously published uncontrolled study demonstrated its feasibility, acceptability, and preliminary effects. This presentation shares findings from a randomized controlled trial evaluating the program’s efficacy using mixed methods (NCT05593276), involving 70 participants aged 60+ recruited from various sources. Participants were randomized to either Empower@Home with lay coach support or to receive attention control friendly calls for ten weeks, with the latter group offered the program after the post-test. High completion rates were noted (89%, Mean = 8.3, SD = 0.4), with significant within-group improvements in the intervention group compared to moderate changes in controls, resulting in a medium between-group effect size favoring the intervention (Cohen’s d =.72, P <.001). Causal mediation analysis showed that acquisition of CBT skills, behavioral activation, and improvements in autonomy, competence, and relationships underpinned these effects. Several themes emerged from a thematic analysis of qualitative interviews with the treatment group: (1) a structured, goal-oriented, and skills-based approach, (2) supportive accountability from weekly coaching, (3) usability and software, (4) a mix of online and physical materials, and personal support to enhance learning, and (5) challenges in personalizing packaged iCBT programs. Empower@Home supported by lay coaches is efficacious in reducing depression among older adults. Implications for the design of digital mental health interventions for older adults are discussed.

